# The AUSTRAL VLBI observing program

**DOI:** 10.1007/s00190-016-0949-y

**Published:** 2016-11-10

**Authors:** L. Plank, J. E. J. Lovell, J. N. McCallum, D. Mayer, C. Reynolds, J. Quick, S. Weston, O. Titov, S. S. Shabala, J. Böhm, T. Natusch, M. Nickola, S. Gulyaev

**Affiliations:** 1 University of Tasmania, Private Bag 37, Hobart, 7001 Australia; 2 Technische Universität Wien, Vienna, Austria; 3 ICRAR/Curtin University, Bentley, Australia; 4 Present Address: CSIRO Astronomy and Space Science, Kensington, Australia; 5 Hartebeesthoek Radio Astronomy Observatory, Krugersdorp, South Africa; 6 Institute for Radio Astronomy and Space Research, Auckland University of Technology, Auckland, New Zealand; 7 Geoscience Australia, Canberra, Australia

**Keywords:** Geodesy, Very long baseline interferometry (VLBI), Celestial reference frame (CRF), Terrestrial reference frame (TRF), AUSTRAL

## Abstract

The AUSTRAL observing program was started in 2011, performing geodetic and astrometric very long baseline interferometry (VLBI) sessions using the new Australian AuScope VLBI antennas at Hobart, Katherine, and Yarragadee, with contribution from the Warkworth (New Zealand) 12 m and Hartebeesthoek (South Africa) 15 m antennas to make a southern hemisphere array of telescopes with similar design and capability. Designed in the style of the next-generation VLBI system, these small and fast antennas allow for a new way of observing, comprising higher data rates and more observations than the standard observing sessions coordinated by the International VLBI Service for Geodesy and Astrometry (IVS). In this contribution, the continuous development of the AUSTRAL sessions is described, leading to an improvement of the results in terms of baseline length repeatabilities by a factor of two since the start of this program. The focus is on the scheduling strategy and increased number of observations, aspects of automated operation, and data logistics, as well as results of the 151 AUSTRAL sessions performed so far. The high number of the AUSTRAL sessions makes them an important contributor to VLBI end-products, such as the terrestrial and celestial reference frames and Earth orientation parameters. We compare AUSTRAL results with other IVS sessions and discuss their suitability for the determination of baselines, station coordinates, source coordinates, and Earth orientation parameters.

## Introduction

VLBI performance for geodesy and astrometry relies on a global network of antennas that are compatible in sensitivity, frequency coverage, and slew speed. The volume of the spanned network correlates with the expected accuracy of measured Earth orientation parameters (Malkin 2009), and uneven antenna distribution makes the results prone to systematic errors (Böckmann et al. 2010). Traditionally, the Northern Hemisphere is much more strongly represented than the Southern Hemisphere. This is the result of long-standing expertise and tradition of this technique predominantly in North America, Europe, and Japan, as well as the geographic land distribution of our planet.

**Fig. 1:**
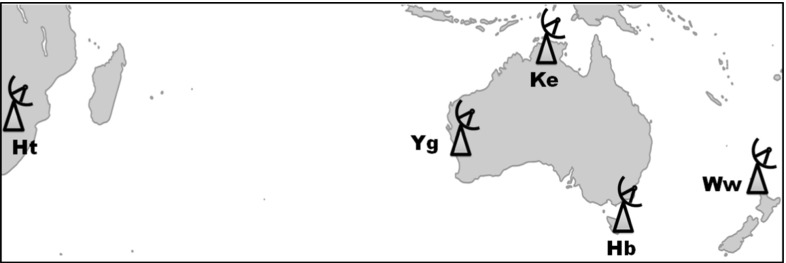
Network of the AUSTRAL stations

Between 1989 and 2011, the only radio telescope observing regularly for geodesy and astrometry in Australia was the 26 m antenna in Hobart (Ho), while the 34 m DSS45 and the 64 m Parkes antennas participated on an occasional basis. Titov (2007) showed that the geodetic performance of the geographically isolated Ho radio telescope was several times worse than those of similar dishes in the Northern Hemisphere, a consequence of the lack of suitable partner antennas. Studying the current antenna networks that regularly contribute to the weekly experiments coordinated by the International VLBI Service for Geodesy and Astrometry (IVS, Schuh and Behrend 2012), we find 15 antennas in the northern and nine antennas in the Southern Hemisphere (Plank et al. 2015b). Five of the nine Southern Hemisphere antennas were built after the year 2010. Three 12 m dishes were erected on Australian territory, in Hobart (Hb), Katherine (Ke), and Yarragadee (Yg). They make up the AuScope VLBI network (Lovell et al. 2013) that is managed and operated by the University of Tasmania (UTAS). The other new antennas are the Warkworth 12 m antenna in New Zealand (Ww), which is practically identical to the AuScope dishes, and a 15 m telescope (Ht) at the Hartebeesthoek Radio Astronomy Observatory in South Africa (Fig. 1).

The IVS is currently in transition to the VLBI Global Observing System (VGOS, Petrachenko et al. 2009), realising a completely new way of observation and operation. The main attributes of VGOS are a much greater number of observations, both in total and per time unit. This shall be enabled by faster antenna slew speeds and shorter on-source times, with the ultimate goal of continuous (24/7) observations. The keys to achieve these goals are (a) building faster and, hence, typically smaller antennas, (b) increasing the recorded bandwidth and frequency distribution to shorten the on-source integration times, and (c) to completely re-think the mode of operation and analysis towards more automation.

The AuScope VLBI array is dedicated to geodetic and astrometric observations and designed for future VGOS compatibility. At present, it is equipped with legacy S/X receivers and state-of-the-art back-end and recording systems (Lovell et al. 2013). However, the fact that the 12 m dishes are less sensitive than most of the other legacy antennas (usually $\ge$20 m in diameter) makes them somewhat inferior in today’s IVS observing program. On the other hand, they have considerably higher slew speeds, allowing for much faster switching between one source and the next. To exploit the full capabilities of the AuScope antennas, the AUSTRAL observing program was initiated. The fact that all three AuScope antennas are remotely operated from a single institution allows more flexibility than in usual IVS operations, triggering the development of new observing techniques. Since 2011, 151 successful AUSTRAL sessions have been observed.

In Sect. 2, the observing program is introduced, highlighting the innovative mode of observation and operation and presenting some general statistics. Since the first AUSTRAL session, this observing program has been the subject to continuous improvements, predominantly as a result of careful optimisation in the scheduling. This is described in Sect. 3. In Sect. 4, we present the results of processing the AUSTRAL data, their suitability for different products is discussed, and the results are validated against those of the standard IVS sessions. The plans for the future of the AUSTRAL observing program are outlined in Sect. 5 and we conclude in Sect. 6.

**Fig. 2:**
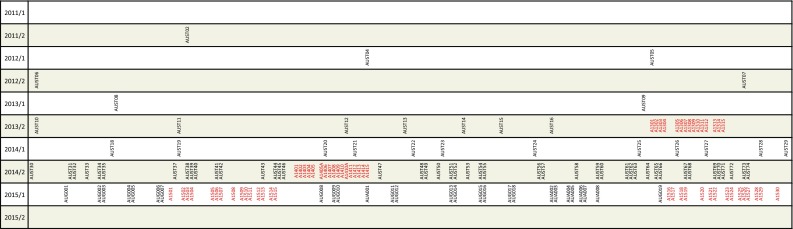
Calendar with AUSTRAL experiments. For each half-year from 2011/2 to 2015/1, the distribution of AUSTRAL sessions is shown on a daily timescale. Sessions of the continuous A-cont campaigns are marked in *red*

## The AUSTRAL observing program

The first experiments that the AuScope telescopes took part in were global IVS sessions in late 2010. This has the big advantage that the coordination of the sessions is facilitated by various components of the IVS. The stations are only responsible for ensuring that they can carry out the scheduled observations and transfer the data to the correlation centre. AUSTRAL sessions were added in 2011. In these sessions, all duties were either directly performed in-house or at least organised by the operations centre in Hobart. As a result, this group gained the expertise in all relevant areas and is now fully and independently capable of planning, scheduling, observing, correlating, and analysing its own VLBI experiments. This is vital for the innovative research on the observation and operation done at UTAS today.

As described below, there are various types of AUSTRAL sessions serving different purposes. Our targets varied between obtaining the most accurate geodetic (baseline) results, a dense time series, or sessions with astronomical purposes. The AUSTRAL program comprises 24 h single sessions, 15 day CONT-like campaigns^1^ and 48 h continuous weekend sessions.

### Overview of experiments

The first successful AUSTRAL session (aust02) was carried out in August 2011. It was scheduled at UTAS and correlated at the Washington correlator. Then, beginning in March 2012 (aust04), the recording mode was changed from the standard IVS mode of 256 Mbps (mega bits per second) to 1 Gbps (giga bits per second). In July 2013 (aust10), a collaboration was arranged with the Technische Universität Wien to deliver the schedules for the AUSTRAL experiments using the Vienna VLBI Software (Böhm et al. 2012; Sun 2013; Sun et al. 2014). At the same time, a contract was signed with Curtin University for correlation using the DiFX software correlator (Deller et al. 2007, 2011). From then on, the post-correlation, including fringe fitting and database creation, was performed at UTAS.

In November/December 2013, the first 15 day quasi-continuous AUSTRAL campaign (A13) was observed (see Sect. 3.1.3). Positive funding and personnel factors allowed the AUSTRAL program to fully evolve in 2014. On average, the AuScope antennas were observing almost every second day of that year, with 72 days dedicated to AUSTRAL sessions. aust31 and aust32 were the first of a series of weekend sessions running on Saturdays and Sundays (Sect. 3.1.3). In September 2014, the A14 campaign was run. Finishing the year with aust74, from 2015, the six-letter code of the AUSTRAL sessions was changed to augnnn for AUSTRAL sessions with a geodetic schedule and auannn for those with mainly astrometric purposes.

High cadence observing continued in 2015 with two 15 day campaigns in February (A15-1) and June (A15-2) and up to aug019 and aua008, before a significant change in operational funding temporarily stopped the AUSTRAL program at the end of June 2015. In addition, the contract for data correlation with Curtin University was not renewed and ten AUSTRAL sessions were correlated at UTAS.

In this paper, we describe the sessions up to June 2015. We note that in beginning of 2016, the AUSTRAL program could be resumed, under collaboration with the Shanghai correlator. Figure 2 shows the distribution of the AUSTRAL sessions on the calendar.

### Statistics

Up to now, there have been 151 AUSTRAL sessions which have produced useful data. More than half of them (81) involved the full five-station network; 42 had four stations, which are usually the three AuScope antennas and either Ww or Ht, and 22 sessions were done with only the three AuScope antennas. In the remaining six sessions, there were good data from only two stations (two sessions) or additional stations were included (four sessions). Table 1 gives the number of AUSTRAL sessions for each of the stations.

**Table 1:** Station contributions to AUSTRAL sessions (2011.5–2015.5)

Station	Hb	Ke	Yg	Ht	Ww
Sessions	151	145	145	103	108

In total, the AUSTRAL sessions comprise about 770,000 observations. One observation means one measured time delay between two antennas (see Sect. 3 for details).

**Fig. 3:**
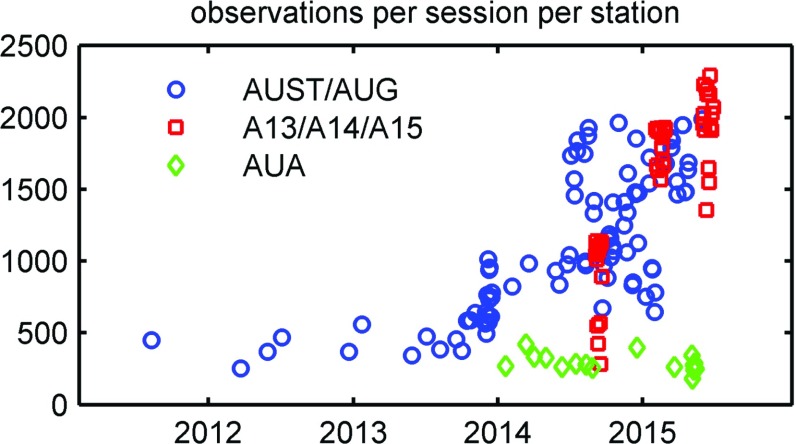
Number of observations per AUSTRAL session as a mean over the contributing stations. It is distinguished between the standard aust/aug sessions (*blue circles*), the quasi-continuous campaigns (*red squares*), and sessions with astrometrical targets (*green diamonds*)

Counting the observations for each antenna, on average, one finds close to 2000 observations per session. As shown in Fig. 3, this number has increased dramatically over time. This is a result of the scheduling improvements described in Sect. 3. For comparison, in a standard global IVS experiment, the number of observations for an AuScope station is between a few hundreds to rarely 1500, depending on the number of contributing antennas.

Perhaps, the biggest problem of the current International Celestial Reference Frame (ICRF2, Ma et al. 2009) is its tremendous inhomogeneity in the number of sources as well as in their positional accuracy between the north and south. It is well known (Ma et al. 2009) that southern sources have nominal source position errors about a factor of two worse than northern sources. It has also been made clear by the same authors that these nominal errors scale with the number of sessions, in which a source has been observed.

In the AUSTRAL sessions, a total number of 357 sources were observed, 220 in the southern sky. 206 of the observed sources have more than 100 observations and 77 sources more than 1000 observations. Considering only sources that were observed in more than 10 AUSTRAL sessions, we find 94 southern and 79 northern targets. In Table 2, these regularly observed sources are binned into areas of declination, showing a distribution that is quite even for the Southern and the (visible) Northern Hemisphere.

**Table 2:** Number of regularly (more than ten sessions) observed sources in the AUSTRAL sessions, for different (absolute) declinations (Dec)

$\left| \mathrm {Dec}\right|$	$0^{\circ }$–$20^{\circ }$	$20^{\circ }$–$40^{\circ }$	$40^{\circ }$–$60^{\circ }$	>$60^{\circ }$
South	38	31	13	12
North	34	31	14	–

It is noted that sources with Dec $>60^{\circ }$ north are not accessible by the AUSTRAL array. On the other hand, Southern Hemisphere arrays are especially needed for observing sources $<-40^{\circ }$, which cannot be observed by common northern arrays (i.e., the VLBA in North America).

### AuScope operations

For the AUSTRAL sessions, we adopted the frequency allocation of the standard IVS R1-experiments, with 8 (6 upper sideband only and 2 upper sideband plus lower sideband) channels in X-band and six channels in S-band. To increase the sensitivity (see Sect. 3), we increased the channel bandwidth from 8 to 16 MHz and used 2 bit sampling instead of 1 bit. As a result, we record more data in one AUSTRAL session, typically up to 6 TB per station per day, than the 2 TB characteristic of a typical R1/R4-session.

The AuScope VLBI array was designed for remote operations, with redundant systems to ensure reliability. All three antennas are remotely controlled by a single operator usually situated at the UTAS campus in Hobart. Much effort has been put into an extensive remote monitoring and control capability. Almost every subsystem is remotely resettable in case communication is lost to that subsystem and the IF chain can be configured remotely. This is even more remarkable given the fact that Yg is connected via an ADSL Internet only, and in Ke, we only have access to a 10 Mbps shared connection through the Charles Darwin University network. For the AuScope antennas, we use a DBBC-2 as the primary data acquisition system with redundant (two at each site) Mark5B+ units for recording. In total, the AuScope array owns and uses more than 100 Mark5 modules, each with capacities of up to 32 TB. All AUSTRAL experiments correlated at Curtin were sent to the correlator via e-transfer. In the case of Ke and Yg, the modules were first physically shipped to Hobart, where the data were transferred from the modules to the local RAID system before it was e-transferred to Curtin. For this, the Hobart observatory is linked to the university campus at 10 Gbps and with 10 Gbps to the Australian mainland.

For observing, a redundant server system was installed which can access the local field system machines, the DBBCs, the Mark5s, and the drive PC. For the observations, we use the eRemoteControl software (Neidhardt et al. 2010a, b) which can be run locally from the observer’s room. The openMoniCA software,^2^ as maintained by the Australian Telescope National Facility (ATNF), is used for extensive monitoring of a multitude of monitor points at each observatory, recording temperatures, motor currents, wind speeds, etc. Operating an array and performing a high number of sessions have also led to a number of new procedures and scripts to ease observation and monitoring: we run an automated data checking procedure performing an autocorrelation for each scan and subsequently display the result. During setup, a fringe check on a strong calibrator is usually done with the AuScope antennas as a way of testing whether all systems are working properly. Enabling identical commands to multiple stations, a multi-operator input program was developed (moprin 3). Finally, a live DBBC formatter time monitoring program has proven to be useful for detecting issues during observations.

In general, at AuScope, we try to distribute responsibilities among staff and observers (typically students). Therefore, we need to log as much information as possible, e.g., concerning the module allocations or data transfers. The pivot of the AUSTRAL operations is the publicly accessible AuScope operations wiki,^4^ a platform for detailed instructions and documentation.

Correlation at UTAS was performed on a six PC cluster with attached multi-TB arrays or, for bigger jobs, the UTAS HPC cluster. In terms of latency, the delay between observation and the preparation of a correlation report was usually several months.

## Scheduling

Scheduling is the process of determining which telescopes observe which source at what time. A schedule is a complex optimisation process which is usually created using a special software. For the AUSTRAL sessions, the newly developed scheduling tool Vie_sched of VieVS (Sun et al. 2014) was used. Vie_sched is to a certain point based on SKED (Gipson 2016), the standard scheduling software used within the IVS. However, while Vie_sched was originally developed mainly for simulations, the AUSTRALs were the first big test for scheduling real observations and hence heavily contributed to further developments. In the following, we briefly describe the basic considerations in scheduling (e.g., Gipson 2016; Sun 2013; Petrov et al. 2009; Gipson and Baver 2016):

When one source is observed by several antennas in a VLBI network, this is usually called a scan. Within one scan, each pair of antennas—a baseline—forms one observation. This means that in a single scan of $n_\mathrm{st}=4$ stations, one usually has $n_\mathrm{obs}=n_\mathrm{st}(n_\mathrm{st}-1)/2=6$ observations. A simple first rule for scheduling is: the more observations the better.

The reality is somewhat more complicated. The largest error in VLBI at present is due to unpredictable fluctuations in the troposphere. To account for this, the general goal is to get as many observations in different directions and at different elevations for each station as possible. This is referred to as *optimising for sky coverage* over a certain period (e.g., 1 h), or simply as the number of scans per hour for an individual station.

In scheduling, both scan length and slewing time are important. An observation can be successfully correlated if the collected signal is sufficiently above the recorded noise (SNR, signal-to-noise ratio). With a target SNR of 15 or 20, the scan duration is then determined by the brightness of the source (flux density *F*) and the sensitivity (SEFD, see Sect. 3.1.1) of the receiving antenna pair (which basically depends on the effective area of the dish and the receiver noise), and the amount of data that is recorded per time unit per band (data rate). For up to 2 bit sampling, the following relationship holds:

1 $$\begin{aligned} t_{\mathrm{scan}}\propto \left( \frac{\mathrm{SNR}}{F}\right) ^2 \times \frac{\mathrm{SEFD}_1 \times \mathrm{SEFD}_2}{\mathrm{data\,\,rate}}. \end{aligned}$$

When developing the new-generation VLBI system VGOS, the key to improving data products was identified as a decrease in the source switching interval (including both slew time and on-source time), meaning observing more sources in a shorter amount of time. As a result, we are seeking shorter slewing times as well as shorter on-source times. While small telescopes are usually able to slew faster than larger ones, their smaller collecting area and hence sensitivity offsets the decreased slewing time with increased integration time. To overcome the sensitivity issue, VGOS aims for a broadband system, allowing to record up to 32 Gbps instead of today’s commonly used 256 Mbps.

In the AUSTRAL sessions, improved source selection, fast antennas, and an effectively four times higher recording rate allowed us to realise a new style of scheduling, somewhere between the present-day approach and VGOS. Typical scan durations of several minutes were reduced down to 20 s, leading to a high of 35 scans per hour per station, compared with about 10 scans per hour in the standard experiments. In the following, we describe some of the key development stages of the AUSTRAL scheduling and how careful work on this topic led to significantly improved results.

### The AUSTRAL scheduling strategy

The realisation of the scheduling in VieVS is described in Mayer et al. (2015). Here, we concentrate on the major steps of improvement of the schedules and outcomes.

#### Antenna capabilities

Besides the scheduling strategy itself, the capabilities of the antennas are decisive. In terms of slewing, the AUSTRAL antennas are fast antennas with slew speeds of up to $5^{\circ }/$s in azimuth and $1.25^{\circ }/$s in elevation (compared with the very fast VGOS antennas of $12^{\circ }/$s in azimuth). Ww has the same speed in azimuth as the AuScope antennas and is a little bit slower in elevation ($1^{\circ }/$s). The 15 m antenna in Hartebeesthoek was scheduled with slew speeds of $2^{\circ }/$s in azimuth and $1^{\circ }/$s in elevation.

The antenna sensitivities are entered into the scheduling as system equivalent flux densities (SEFD) measured in Jansky (Jy). According to Lovell et al. (2013), the AuScope antennas with room-temperature receivers have zenith SEFDs of 3500 Jy in X-band and 3400 Jy in S-band, degrading to about 4500 Jy at low elevation. So far, elevation dependency of the antenna SEFD has not been considered in the scheduling of the AUSTRAL sessions. In Table 3, the SEFDs used for each antenna are shown, together with the dates from which these were applied.

In the beginning, rather conservative values of about 5000 Jy were used. These values were determined with the help of colleagues from the Goddard Space Flight Centre based on the analysis of recent observations. For Yg, a bit higher values were used, accounting for hardware problems leading to failures in various channels. In October 2014, the AUSTRAL program started to maintain its own catalog files and then beginning with aust50 the S-band SEFD at Hb was increased to 5300 Jy to account for a large amount of S-band radio frequency interference (RFI).

The last change in antenna SEFD followed a thorough analysis of the A14 observations, with new SEFDs from a1501 onwards. At the moment, Yg shows the best performance, with a sensitivity of 3600 Jy in X-band. This is close to the theoretical value of 3500. The expectations for Ww were significantly improved after a major service of the DBBC. On the other hand, the values for Ht were found to be a little too optimistic and increased to 2200 and 1400 Jy in X- and S-bands, respectively.

**Table 3:** Antenna sensitivity used in the scheduling of the AUSTRAL sessions

X-band	Hb	Ke	Yg	Ww	Ht
aust02	5000	4500	9000	–	–
aust03		5000	5000	5000	–
aust07			9000		–
aust08		4500			–
aust09				8000	–
aust10					1400
aust18					
aust50	4300	4800	5000		
aust56					
a1501	4600	4200	3600	4600	2200

S-band	Hb	Ke	Yg	Ww	Ht
aust02	4000	5000	5500	–	–
aust03		4000	4000	4000	–
aust07			5500		–
aust08		5000			–
aust09				8000	–
aust10					1050
aust18			5000	5000	
aust50	5300	4800	4300	8000	
aust56				5000	
a1501	5000	5200	4000	4200	1400

Values are given for sessions, where changes were made. In between, the previous SEFD value is used. SEFDs for both X-band (upper table) and S-band (lower table) are provided in Jy

It should be noted here that determining the antenna-specific SEFD, a posteriori of observations within a network, is not straightforward. A recent study by Gruber (2016) revealed considerable variations in those values for the AuScope antennas and suggests that more research is needed.

**Fig. 4:**
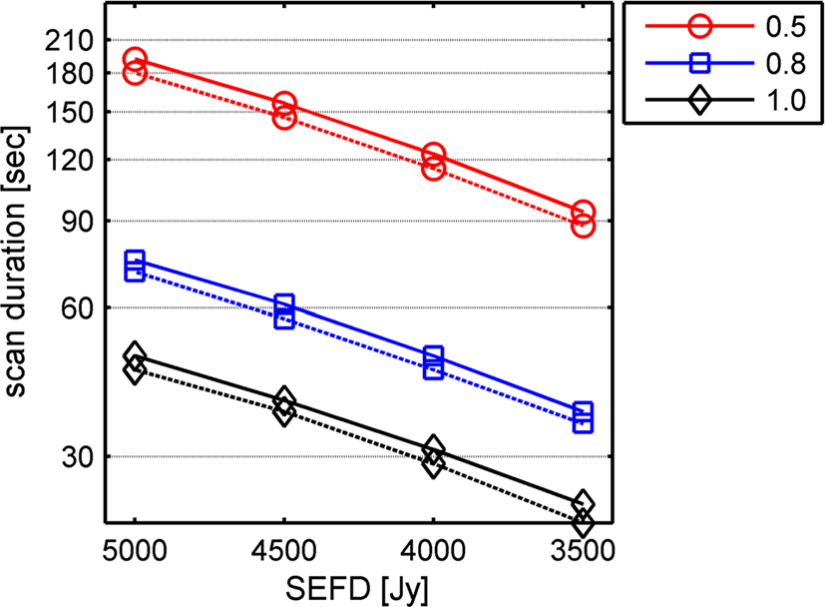
Scheduled scan lengths as a function of antenna SEFD. Two antennas of identical SEFD of 3500, 4000, 4500, and 5000 Jy were assumed. Scan lengths were calculated for a source with flux density of 0.5 Jy (*red circles*), 0.8 Jy (*blue squares*), and 1.0 Jy (*black diamonds*). Using the AUSTRAL recording mode, the *solid line* represents the limit for a minimal SNR of 20 in X-band and the *dashed line* the S-band limit for a minimal SNR of 15

To understand the effects of changing the antenna SEFDs in the scheduling, in Fig. 4, the interaction of antenna sensitivity and scan duration is shown for three different sources with flux densities of 0.5, 0.8, and 1.0 Jy.

Using the AUSTRAL observing mode (data rate of 1 Gbps) for a relatively strong source of 1.0 Jy, the scheduled scan duration changes from 24 s for two antennas with SEFD of 3500 to 31 s when both antennas have SEFDs of 4000, and up to 48 s for SEFDs of 5000. For weaker sources of 0.8 Jy flux density, the scan duration is 37 and 75 s for SEFDs of 3500 and 5000 Jy, respectively, and for a source of flux densities of 0.5 Jy, the scan duration varies between 94 and 192 s for the same respective SEFDs.

#### Schedule development

Scheduling of AUSTRALs evolved for three reasons:

- we used completely new software which was originally designed for simulations and was not fully verified for real observations. When applying VieVS for the scheduling of AUSTRAL sessions, a number of new features and scheduling options were added to the software;
- the schedulers themselves were quite inexperienced in the beginning and they are still learning and improving their understanding of the scheduling process;
- the AUSTRAL sessions allow for fast slewing and short on-source times (in the style of the future VGOS), demanding for new scheduling that differs from that for the legacy experiments.

**Fig. 5:**
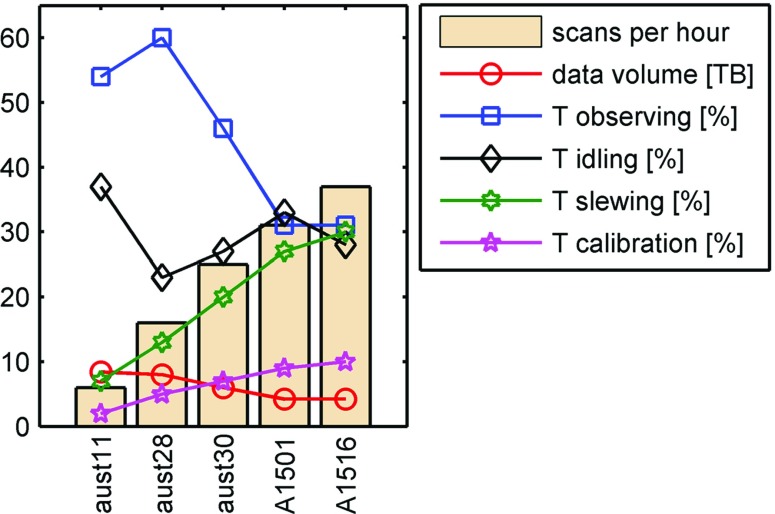
Key schedule parameters and their development for different AUSTRAL sessions. All values are means over five participating antennas. The *bars* in the background indicate the number of scans per hour. The *blue symbols* show the percentage of time that a station dedicates to observations (*blue squares*), slewing (*green hexagons*), calibration (*magenta stars*), and idling time (*black diamonds*). The *red circles* show the amount of data per station in TB

In Fig. 5, the development of the key parameters of a schedule summary is shown. We only picked sessions, where all five AUSTRAL stations were scheduled, as well as sessions, where effective changes to the scheduling strategy were applied. On the very left, we find the parameters of aust11, a schedule in the style of the legacy observations. On average, six scans per station per hour are done. An antenna spends 54 % of time on observations and 7 % on slewing. For 37 % of the session an antenna is idle, meaning that it is not used and presumably waiting for other antennas. This is mainly due to the inclusion of Ht. While the three AuScope antennas and Ww are practically identical and have similar capabilities, Ht is much more sensitive but also slower in slewing. In addition, the mutual visibility between Ht and Ww is limited, causing some difficulties in scheduling. For aust11, an additional 2 % of the time was spent for calibration, which are fixed-time intervals added at the beginning and the end of a scan. For the first sessions, data of up to 8.4 TB were recorded per station.

As already mentioned above, the VGOS goal is to increase the number of scans for a station. We adopted this goal for the scheduling of the AUSTRAL sessions. A major step was taken in aust30, restricting the source list to sources stronger than 0.5 Jy. Later on (starting with a1501), using simulations (in the VGOS style; Petrachenko et al. 2009; Pany et al. 2010), we found that a cutoff at 0.8 Jy gives the best baseline length repeatabilities. This new cutoff led to an increase in the number of total observations from $\sim$4000 (cutoff 0.5 Jy) to $\sim$5000 in the full AUSTRAL network and an increase in the mean number of scans per hour per station from 23 to 30. Further scheduling improvements were achieved by increasing the weight for observations of the Ht station, accounting for the low visibilities due to the network geometry. All the AUSTRAL schedules were generated in an iterative process, excluding sources with a low (<4) number of observations, and down-weighting sources with too many observations. With the relative observing time decreasing from about 60 to 30 %, we found the times accounting for calibration becoming more and more important. As a consequence, some of these times were drastically shortened in our scheduling.

For the final A15-2 series (starting with a1516, see Sect. 3.1.3), we have an average number of 37 scans per hour per station. This means that our antennas spend about as much time on source for observations, as they need for slewing. With the remote location and the different capabilities of the Ht antenna, on average, there is also 30 % of the time spent idling. The calibration time increased to 10 % of the total time, indicating that further optimisation in the actual times needed for the recorder to switch on or to allow for automatic measurements of the system temperatures in the field system is worthwhile. This includes improvements in the station-specific procedures to ensure that non-essential checks do not delay scheduled commands in the field system. Finally, with less time spent on actual observing, the total data recorded at a station over the entire session of 24 h duration was halved from $\sim$8 to $\sim$4 TB.

Using the commonly used method of baseline length repeatabilities, studying the results of the AUSTRALs using different generations of the scheduling procedure, a clear improvement was found. This will be discussed in more detail in Sect. 4.

#### Weekend and AUST-CONT experiments

The weekend and continuous AUSTRAL sessions have some special scheduling features which are described here briefly.

Standard IVS sessions usually start at a certain time (e.g., 17:00UT) and run for 24 h. This means that the last scan of a session starts within this 24 h and often finishes a couple of minutes after 17:00UT the next day. This would cause a problem when scheduling two consecutive sessions. For the AUSTRAL weekend sessions starting at 00:00UT on a Saturday and Sunday, the first session was, therefore, scheduled to be 5 min short of 24 h. In addition, the schedule file (.snp) of the first session was edited to start the following session, allowing for an automatic session changeover without additional interaction by the operator.

Within the AUSTRAL program, four quasi-continuous AUST-campaigns of 15 days observing were performed: A13 in end of 2013, A14 in September 2014, A15-1 (a1501-a1515) in February, and A15-2 (a1516-a1530) in June 2015. We use the term quasi-continuous as the 15 24 h sessions were placed around other observing commitments, such as the IVS R-sessions which occur twice a week. This, on the other hand, allows for a nice comparison of measured parameters between the different types of sessions (see Sect. 4).

A special feature of the A-cont campaigns is that the same schedule was repeated every sidereal day (also see Mayer et al. 2015). This guarantees that, for a given observation, the relative angle between the observing baseline and source structure direction (i.e., the quasar jet projected in the plane of the sky) remains fixed from day to day. As a consequence, we expected the systematic effects of quasar source structure (e.g., Shabala et al. 2015; Plank et al. 2016) to be the same each day and, hence, to not affect repeatabilities of results during one campaign. For A13, we went even further, repeating two different schedules: one with only good sources (or a nominal structure index (SI, Fey and Charlot 1997; Ma et al. 2009) smaller than 2.5) and a second schedule with only bad sources with SI higher than 2.5. We note that quasar structure is not expected to vary significantly on the relatively short timescales of $\sim$2 weeks (Shabala et al. 2014; Lister et al. 2009). However, the analysis, so far, revealed that other errors are larger than those produced by source structure. The investigation is ongoing.

The sheer fact that these A-cont sessions spread over a few weeks provides a unique record of almost continuous VLBI observations for such a long period. In June 2015, the AuScope antennas observed on 25 of 30 days of that month. This opens up for the first-time opportunities, such as a fair comparison with the continuously operating antennas of the GNSS for a longer time period than the usual 15 day IVS VLBI Cont Campaigns.

### Scheduling of astro-sessions

An exception from above-mentioned scheduling developments is the AUSTRAL-astro-sessions. The main target of these experiments were the observations of special sources, which were not regularly observed elsewhere. This included sources observed for the first time in dual-frequency mode, with the goal to identify new strong sources in the southern sky suitable for geodetic/astrometric VLBI. A list of these sources is given in Sect. 4.6. Some of the AUSTRAL-astro-sessions were used for other purposes: for example, to perform observations to a source very close to the Sun, to study the effects of general relativity (Titov and Girdiuk 2015). For more information, the reader is referred to Mayer et al. (2015) and Plank et al. (2015c).

As is clearly visible in Fig. 3, the astro-sessions (marked with green diamonds) have typically far fewer observations than the geodetically optimised experiments. This is due to the fact that a different list of sources was used and that weaker sources requiring longer scan lengths are observed. With the AuScope array, sources down to 0.4 Jy flux densities can be observed with scan lengths up to 500 s. For several of these astro experiments, additional larger dishes, such as the Hobart 26 m, the Parkes 64 m, or Tidbinbilla 70 m dish, were included to increase the sensitivity. An improved scheduling strategy for these combined, small low-sensitivity dishes observing with one or more large, highly sensitive dishes is currently under development.

## Results

### Data

In this study, we use the data from 151 AUSTRAL sessions, which is largely publicly available via the IVS. For comparison, we processed the standard IVS sessions (Nothnagel et al. 2015) for the same time period, from 1/2011 to start of 11/2015. These standard sessions are the rapid-turnaround (R-) experiments that are observed twice a week (R1 on Mondays and R4 on Thursdays) with a global network of around nine antennas (median). These IVS R-sessions are a strong contributor to the standard IVS products, such as EOPs, TRF, and CRF.

### Processing

Data were analysed using the VieVS software version 2.3, as maintained by the Vienna group. This incorporates all standard analysis models, in accordance with the IERS Conventions (Petit and Luzum 2010). For a priori information on the coordinates, the Vienna contribution to the VTRF, VieTRF14 (Krásná et al. 2014), was used. Station positions were estimated once per session and the datum was set via a no-net-translation (NNT) and a no-net-rotation (NNR) condition on the stations of this TRF. This means that all AUSTRAL stations were datum stations. For the CRF, all sources were also estimated once per session, with the datum set on the defining sources of the ICRF2 (Ma et al. 2009) using an NNR condition. Troposphere zenith wet delays were estimated as piecewise linear offsets every 30 min using loose constraints of 1.5 cm over 30 min. The estimation interval for atmospheric gradients was set to 3 h using loose constraints as well. Station clocks were estimated once every 60 min. The observations were weighted using the inverse of the sum of squared formal uncertainties and, as these tend to be too optimistic, adding an additional noise floor of 1 cm$^2$; this yields realistic uncertainties (Plank et al. 2015b; Shabala et al. 2015)

When determining EOPs (Sect. 4.5), all sources were fixed to their catalog positions. For the estimation of source positions (Sect. 4.6), a global solution was performed for each set of sessions. By stacking the normal equation matrices of all sessions into a single least-squares adjustment, a set of global station and source coordinates was estimated. Again, all defining sources of the ICRF2 were used as datum sources, realised with an NNR plus dz-condition. Sources which were observed in less than five sessions were reduced in the analysis.

### Session performance

One way to quantify session performance is to count the number of successfully correlated observations. For the AUSTRALs, we find a median 88 % of successfully correlated observations from those originally scheduled. Reasons for unsuccessful observations are insufficient SNR or simply missed scans due to a wind stow or other technical issues. In 28 sessions, there were more severe problems, e.g., a complete station failure, and the percentage of successfully correlated scans drops to below 60 %.

**Fig. 6:**
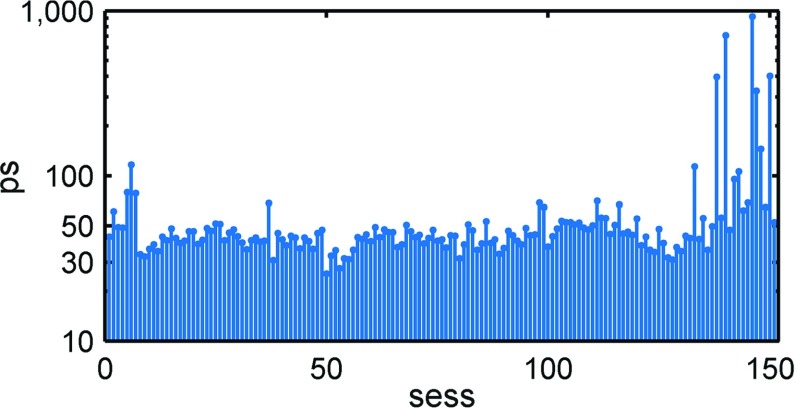
Post-fit residuals (rms) of the AUSTRAL sessions. The sessions are ordered with time

Next, we can look at the post-fit residual delays after processing. These are shown in Fig. 6 as rms values over all observations of a session.

We find a median session fit of 44 ps over all sessions. This is considerably worse than the IVS R1/R4 observations with a median value of 34 ps. The reason for this is not completely understood yet. Studying Fig. 6, we find large residuals in the beginning of the AUSTRAL program as well as for the latest sessions, where we had severe problems with multiple clock jumps at the AuScope stations which could not be handled properly in the analysis. This problem was identified as being caused by high voltages induced to the maser by high currents in the compressor of the air conditioning unit located in the same room. After a major maser maintenance in 2016, measures against this coupling have been installed. It is also evident that the AUSTRALs did show an improved session fit of around 30 ps over two periods, once in August 2014 just before A14 and then again in April 2015 (aug013-aug018). The reasons for this exceptionally good behaviour could not be identified so far.

One possible reason for the generally worse residuals is that due to strong RFI in two S-band channels at Hb, these data are usually omitted from correlation. Consequently, the broadband resolution function is wider, leading to higher group delay errors in S-band. Although the S-band is only used to calibrate the ionosphere and errors are usually heavily reduced (by a factor^5^ of 1/13, Porcas 2010), we find a considerable number of very low SNR and non-detections in S-band. To overcome this, when resuming the AUSTRAL program in 2016, the target SNR in S-band was raised from 15 to 20, leading to longer scan lengths. The first session, aug020, shows an improved session fit of 26 ps.

### Antenna positions and baselines

**Fig. 7:**
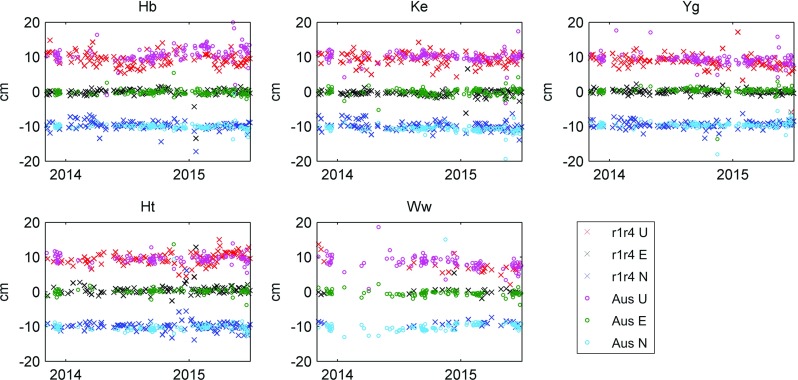
Antenna position estimates. For each station, the estimated residuals to the catalog coordinates are shown in Up (*red* and *magenta*), East (*black* and *green*), and North (*dark and light blue*) direction. For better visibility, the Up and North components are offset by 10 cm. Results are shown for the time period between 11/2013 and 7/2015. In the case of Hb, there are 138 AUSTRAL sessions (results displayed with *circles*) and 107 R1/R4 sessions (*crosses*) in this period

In our analysis, antenna positions were estimated once per session. In the processing, the station’s movements are modelled to the best a priori knowledge, including station velocities and geophysically induced variations (Petit and Luzum 2010, Chapter 7), and the estimated residuals are the offsets to the catalog positions. In Table 4, the mean offsets and the standard deviations are given for each of the stations, split in the local Up-, East-, and North components. It is distinguished between the processed AUSTRALs and the IVS R-sessions. Figure 7 allows a visual interpretation of the results, showing a subset of sessions between 11/2013 and 7/2015.

**Table 4:** Statistics of the estimated residuals in station positions (Up, East, North) with respect to the catalog positions

	Up (cm)	East (cm)	North (cm)
Hb			
Aus	0.8 ± 1.6	−0.1 ±0.6	0.0 ±0.7
R1R4	−0.4 ± 2.0	0.0 ±0.8	0.1 ±1.3
Ke			
Aus	0.2 ± 1.0	0.0 ±1.0	−0.6 ±0.8
R1R4	−0.2 ± 1.6	−0.1 ±0.8	0.0 ±1.2
Yg			
Aus	−0.8 ± 1.0	0.4 ±0.6	0.5 ±0.8
R1R4	−1.0 ± 1.8	0.1 ±0.8	0.3 ±1.1
Ht			
Aus	−0.4 ± 1.3	0.1 ±0.7	−0.3 ±0.8
R1R4	−0.3 ± 1.4	0.5 ±1.0	0.0 ±1.1
Ww			
Aus	−1.4 ± 1.5	−0.4 ±0.8	0.1 ±1.0
R1R4	−2.5 ± 2.3	0.0 ±1.0	0.7 ±0.9

For each of the five stations, the mean offset (first number) and the standard deviation (second number) are given, calculated over all processed AUSTRAL, respectively, R-sessions

For session-wise estimated station positions, we find offsets up to 1–2 cm. In general, the results of the regional AUSTRALs and the global R-sessions agree well. Looking at the statistics, we find the AUSTRAL results to be less noisy. This is presumably due to the fact that the network of the global R-sessions varies, having different datum stations for each session in the session-wise solution. For both types of sessions, we find significant mean offsets up to the centimeter level. This indicates poor a priori station coordinates, most likely a result of the relatively short observing history of the AUSTRAL stations.

If present, the estimated offsets of both session types follow similar patterns (Fig. 7): there seems to be a periodic signal in the height component at the Hb station of $\pm 2$ cm. This may also explain the large discrepancy between the two solutions in the estimated mean Up component for Hb. For comparison, analysing data between 2003 and 2013.3 Krásná et al. (2015) find a seasonal signal of about ±5 mm for the co-located Hobart 26 m antenna. The signal for the Hobart 12 m antenna is even larger and also present in the local 12–26 m Hobart baseline (Plank et al. 2015a). The reason is not yet clear and needs further investigation. The dense time series also reveals a sudden subsidence of about −2 cm in the height component of Ww at the beginning of 2015. Here, both solutions indicate similar results.

We can conclude that the regional AUSTRAL sessions are very suitable for the determination of absolute station coordinates. In addition, the high cadence of these sessions is of great benefit for studying unmodelled station movements.

**Fig. 8:**
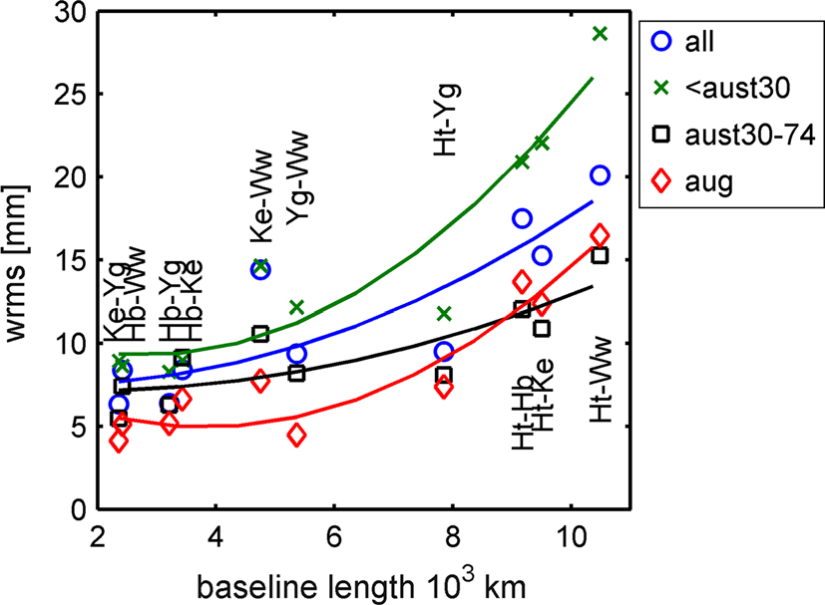
Baseline length wrms of different sets of AUSTRAL sessions. We distinguish between all AUSTRAL sessions (*blue circles*), sessions before aust30 (*green crosses*), from aust30 to aust74 inclusive (*black squares*), and the most recent aug sessions (*red diamonds*). For better visibility, a second-order polynomial was fitted through the different baselines

Next, we discuss baseline lengths and their repeatability. With VLBI being a relative technique, it is ultimately sensitive to the relative distance between the network stations, their baseline lengths. Comparing the baseline lengths as determined in different sessions is a well-established method in the community to quantify the precision or repeatability of the measurements. As discussed above, all known station movements are modelled in the processing and one would not expect the estimated baseline to change. Baseline lengths repeatabilities were then calculated as the wrms of the session-wise estimated lengths. In Fig. 8, the repeatabilities are shown for different sets of AUSTRAL sessions: all sessions up to aust30, including the A13 campaign, then results for aust30-74, including A14, and finally, the aug, aua, and A15 sessions. As described above (Sect. 3), this classification is based on major changes in the scheduling.

**Table 5:** Baseline length repeatabilities (wrms) as determined in the AUSTRALs, various subsets of them, and the R1/R4 sessions from 1/2011 to 11/2015

Baseline	Length (km)	All (mm)	(151)	<aust30 (mm)	(40)	aust30-74 (mm)	(54)	aug (mm)	(49)	R1/R4 (mm)	(494)
Ke–Yg	2360	6.3	(139)	8.9	(36)	5.5	(52)	4.1	(44)	8.6	(175)
Hb–Ww	2416	8.4	(108)	8.6	(30)	7.4	(42)	5.1	(34)	7.9	(33)
Hb–Yg	3211	6.4	(145)	8.3	(38)	6.3	(52)	5.2	(47)	9.0	(154)
Hb–Ke	3432	8.4	(145)	9.0	(38)	9.1	(54)	6.7	(46)	13.0	(161)
Ke–Ww	4753	14.4	(105)	14.6	(28)	10.6	(42)	7.7	(33)	18.5	(48)
Ww–Yg	5362	9.4	(106)	12.2	(28)	8.2	(42)	4.5	(34)	13.2	(44)
Ht–Yg	7849	9.5	(99)	11.8	(29)	8.1	(31)	7.4	(35)	14.8	(108)
Hb–Ht	9167	17.5	(103)	20.9	(29)	12.0	(33)	13.7	(37)	17.8	(83)
Ht–Ke	9504	15.3	(99)	22.0	(27)	10.9	(33)	12.4	(36)	16.8	(104)
Ht–Ww	10481	20.1	(81)	28.7	(22)	15.3	(28)	16.5	(30)	14.7	(35)

Results are shown for the baselines of the AUSTRALs, including the antennas at Hobart (Hb), Katherine (Ke), Yarragadee (Yg), Hartebeesthoek (Ht), and Warkworth (Ww). The number of sessions, in which a baseline was observed, is given in brackets

Results in terms of baseline repeatabilities have improved by almost a factor of two since the beginning of the program. The short baselines (up to 3500 km) have been improved from about 9 mm to between 4–7 mm in baseline length repeatability. For the very long baselines, we find improvements from 2–3 to 1.5 cm for the most recent sessions. Studying Fig. 8, in more detail, we find almost continuous improvements with scheduling development, with the exception of the very long baselines to Ht after the most recent change. Although this needs further investigation, a possible explanation is that with the scheduling change at the beginning of 2015, the nominal antenna SEFD for Ht was drastically increased (Table 3). This would lead to longer on-source times and consequently to less observations for Ht. On the other hand, there have been some efforts trying to upweight observations to Ht more recently (this is the main difference between the schedules for a1501 and a1516, as shown in Fig. 5), and we hope to improve the results on these baselines again. The different treatment of Ht may also explain the quite different shape of the fitted polynomials in Fig. 8: while the initial (<aust30) and most recent (aug) sessions quickly rise for the long baselines with Ht, the curves are much flatter for the other two sets of data. Given the fact that the reason for the curvature seen in baseline repeatabilities is a point of discussion (Titov 2009), this might be of interest.

The individual values of baseline wrms are summarised in Table 5.

Each set of AUSTRAL sessions in our comparison has roughly the same number of sessions ($\sim$30) allowing for decent statistics. We also give the numbers for the processed R-sessions. All baselines show better results in the AUSTRALs. The single exception is the baseline Ht–Ww, where the R-sessions show slightly better performance (14.7 mm compared to 20.1 mm—all sessions—or 16.5 mm—aug sessions). Despite these impressive improvements over the two years, results of the IVS Cont14 series are better still, especially on the long baselines (see the comparison in Plank et al. 2015c). The Cont14 series incorporated a 512 Mbps recording mode, which is between the current R-sessions and the AUSTRAL mode, and observed with a global network of 17 antennas. We believe the reason for the differences is the fact that Ht is much better connected in the global network, leading to a higher number of observations ($\sim$1800) compared with $\sim$1400 in the latest AUSTRAL sessions.

### Earth orientation parameters

VLBI is the only space geodetic technique capable of measuring all five Earth orientation parameters (EOPs). However, it has been shown (Malkin 2009) that both the accuracy and precision of measured EOPs are strongly correlated with the size of the observing network, or more specifically, the volume *V* of its spanned area on the globe. The maximum volume of the AUSTRAL sessions is 18.7 $\mathrm {Mm^3}$ (cubic megametres) compared with a median of 222 $\mathrm {Mm^3}$ for the R-sessions. On the other hand, it has also been shown that the EOP accuracy and precision also depend on the recording rate, though to a lesser extent than the network volume (Malkin 2009).

In Table 6, we show the calculated accuracy of the EOPs for the investigated sessions. The accuracy was determined as the median absolute estimates, representing the median offsets to the a priori C04 08 series. As a second quantity, the median formal errors are given, representing the assumed precision of the estimates.

**Table 6:** Nominal accuracy and precision for the Earth orientation parameters calculated as the median absolute estimates and the median formal errors

	Pole *x* ($\upmu$as)	Pole *y* ($\upmu$as)	dUT1 ($\upmu$s)	dX ($\upmu$as)	dY ($\upmu$as)
Aust	$434\pm 244$	$812\pm 298$	$39\pm 17$	$116\pm 75$	$139\pm 75$
R1R4	$115\pm 72$	$195\pm 78$	$10\pm 4$	$47\pm 37$	$66\pm 36$

Values are given for sessions-wise estimates of the AUSTRAL sessions and the analysed R1/R4 experiments

We find that the AUSTRAL sessions are not very well suited for determining polar motion. While the results from the R-sessions deviate about 100–200 $\upmu$as from the a priori values, the estimated offsets for the AUSTRAL sessions are four times larger. A median offset of about 800 $\upmu$as in *y*-pole with a formal uncertainty of 300 $\upmu$as shows that the AUSTRAL network is simply not sensitive enough in this direction. Given a considerably longer east–west baseline, one might expect better results for estimates of dUT1. Results do look better than for polar motion, but they are still much worse than for the R-sessions (39 versus 10 $\upmu$s in median offsets). The differences between the two types of sessions become smaller for precession/nutation dX/dY. Here, the AUSTRAL sessions are only twice as bad as the experiments using a global network.

**Fig. 9:**
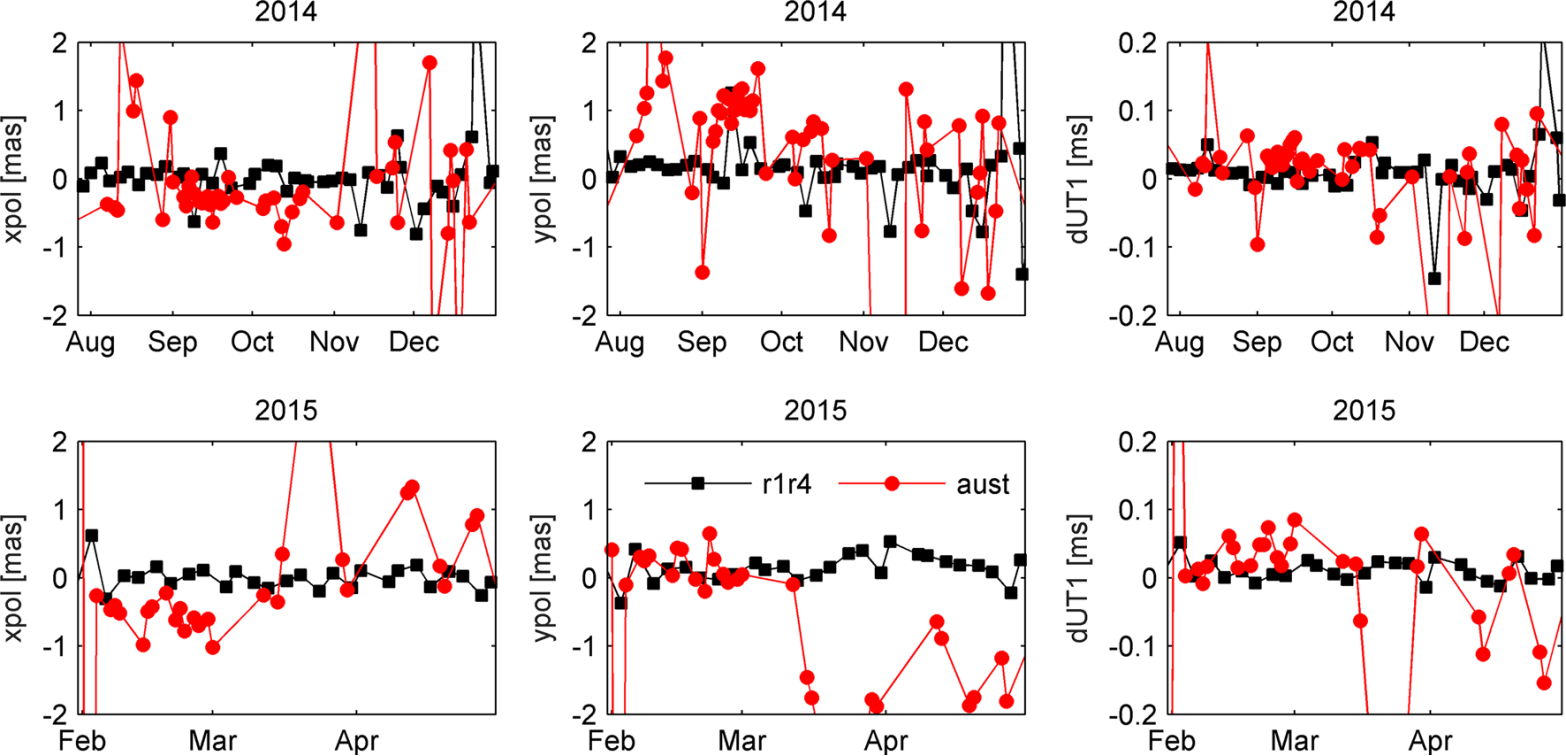
Comparison of estimates for Earth rotation parameters between the regional AUSTRAL (*red circles*) and the global R-sessions (*black squares*). The *top row* shows sessions between August and December 2014; the *bottom row* gives results between February and April 2015. Estimates are residuals to the IERS C04 08 series

Despite the poor statistics for the AUSTRAL sessions in Table 6, there seems to be some potential for these sessions to produce useful measurements of the EOPs. In Fig. 9, we compare the results of the regional and global sessions for two periods of intense observing.

We find that for some sessions and even a consecutive set of experiments, the agreement with the R-sessions is remarkably good, while for other sessions, there seems to be a systematic offset. There is good agreement for *y*-pole and to some extent also *x*-pole and dUT1 for the AUST-CONT A15-1 series in February 2015, while the following sessions show large, almost systematic offsets. This also partly applies to the April sessions, which show particularly good session fits (see Fig. 6). In the second half of 2014, we find erratic behaviour, with one session showing good agreement and the next being considerably off. This also applies to some of the weekend sessions, revealing considerably different results between the Saturday and Sunday sessions. This indicates that the performance of the session, meaning how many successful observations or whether a station had problems, is really decisive for the EOP results. Attempts to correlate the station network (only AuScope, AuScope+Ww, AuScope+Ht, AuScope+Ht+Ww) with EOP accuracies did not reveal any clear trends.

In summary, one can say that at the moment, the measurement of EOPs in AUSTRAL sessions shows, on average, considerably worse results than the global R-sessions. On the other hand, there are some periods with much lower rms EOP scatter, although still having significant bias from the R-series. To identify clear reasons for these highly varying results, more work will be necessary.

Another area of high potential of the AUSTRAL sessions may be high-frequency EOPs. Without having performed the necessary studies yet, it can be assumed that the uniquely high cadence of the VLBI time series of the combined AUSTRAL and standard IVS experiments may offer new possibilities for the determination of sub-daily EOPs with VLBI.

### Source positions

**Fig. 10:**
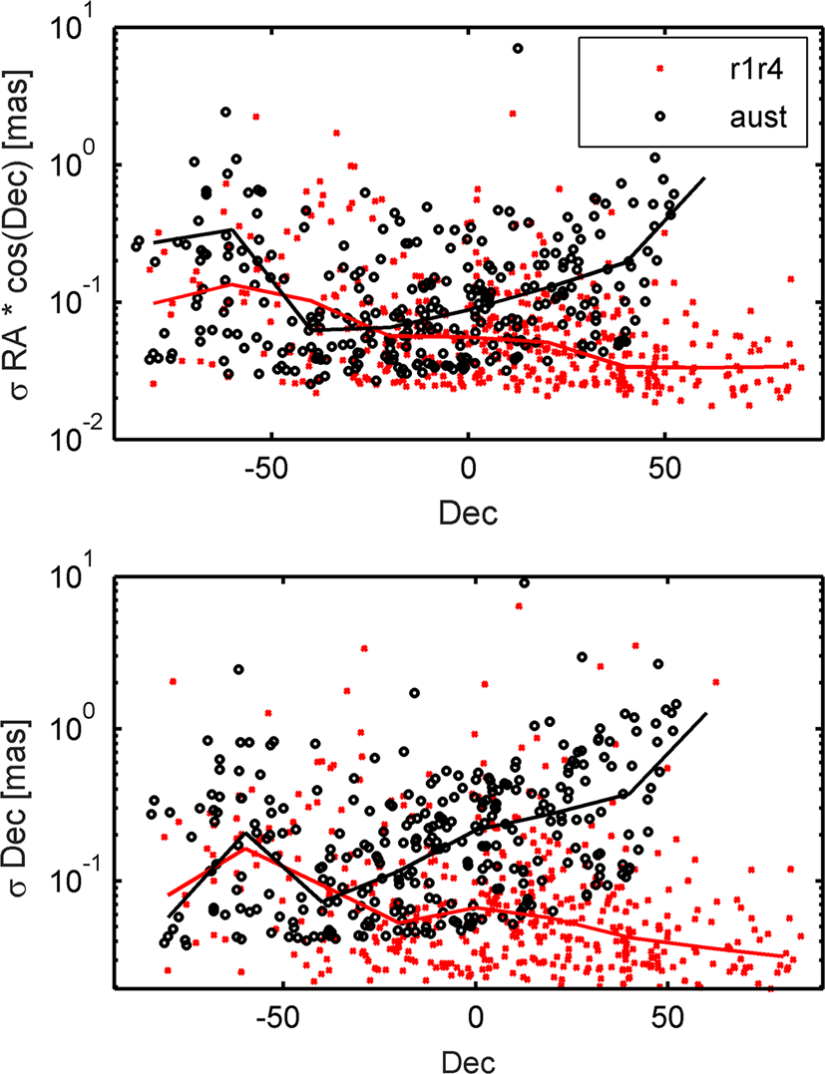
Formal uncertainties in right ascension (*top*) and declination (*bottom*) versus declination of sources estimated within a global solution. Results are shown for the global R1/R4-sessions (*red crosses*) and for the AUSTRAL sessions (*black circles*). The *solid lines* are the median formal uncertainties calculated over bins of $20^{\circ }$ in declination. The formal uncertainties are given in mas on a logarithmic scale

As a last topic, we discuss the ability of the AUSTRAL sessions to determine source positions. As already mentioned in the introduction, the observed sources within the AUSTRAL sessions are homogeneously distributed over the visible sky, reaching Dec = $60^{\circ }$ north. To facilitate comparison between the AUSTRALs and the R-sessions, two individual global solutions were performed using identical datum definitions (see Sect. 4.2). In Fig. 10, the formal uncertainties ($\sigma$) are shown for right ascension (RA) and declination (Dec). The errors on RA are scaled with cos(Dec).

There is a wide range of uncertainties found amongst the observed sources. At best, we find uncertainties of 25 and $20~{\upmu }as$ for the R-sessions in RA and Dec, respectively. These values are slightly worse for the AUSTRALs, with a lower limit (=best values) of about 30 and $40~{\upmu }as$ in RA and Dec. Looking at the RA, it becomes obvious that the strength of the AUSTRAL sessions is in the estimation of sources right above the network, between $-20^{\circ }$ and $-50^{\circ }$ declination. Here the formal uncertainties are better than those of the R-sessions. On the other hand, the median uncertainties south of $-50^{\circ }$ are worse for the AUSTRAL sessions than for the R-sessions, the origin of which is not fully understood. The errors of sources in the Northern Hemisphere are also clearly worse than in the global sessions. Concerning declination, the errors of the AUSTRAL sessions are comparable with those of the R-experiments for southern sources of about $-20^{\circ }$ and south, and quickly degrading for sources with declinations further north.

In terms of source positions, we do not find any systematic differences between estimates of the two different types of sessions.

Overall, there are 208 sources observed in common in the two types of sessions. There are also 91 sources regularly observed in the AUSTRALs, which were not targeted in the investigated R-sessions. 76 of them can be determined to better than 1 mas and 31 to even better than 200 $\upmu$as.

Table 7 lists the target sources of the AUSTRAL-astro-program. Most of them were observed in between 2–8 AUSTRAL sessions and show positional formal uncertainties of $\sim$300 $\upmu$ as in RA and Dec for single-session solutions. Those sources were chosen due to their limited number of observations (as per ICRF2 status) but are strong enough (flux densities of typically >0.4 Jy) to be detected by the network of 12 m telescopes.

**Table 7:** Target sources of the AUSTRAL-astro-program

Source	Type	Flux S	Flux X	sess
0002-478	Def	0.2	0.2	2
0056-572	Non-def	0.3	0.2	5
0048-427	Def	0.3	0.4	2
0107-610	Def	0.25	0.3	2
0122-003	Non-def	0.4	0.3	8
0142-278	VCS	0.5	0.4	5
0230-790	Def	0.35	0.45	34
0312-770	Non-def	0.3	0.3	2
0742-562	Single F	0.4	0.4	2
0743-673	Single F	0.4	0.7	8
0758-737	Single F	0.2	0.1	18
1030-590	Single F	0.4	0.1	1 (7 n/d)
1312-533	Single F	0.4	0.4	5
1319-093	VCS	0.4	0.7	5
1336-237	VCS	0.5	0.4	8
1352-632	Non-def	0.3	0.14	n/d
1511-360	Single F	0.35	0.5	2
1531-352	Non-def	0.5	0.4	8
1707-376	Single F	0.4	0.19	n/d
1740-517	Non-def	0.4	0.4	5
1842-289	VCS	0.2	0.3	8
1922-224	Non-def	0.22	0.17	6
1933-400	Def	0.6	0.9	26
2030-689	Non-def	0.3	0.7	6
2354-117	VCS	0.7	0.25	9
2355-534	Def	0.7	1.5	70

We distinguish between defining and non-defining sources of the ICRF2, sources of the VLBA calibrator survey (VCS), and sources which have only been observed in single-frequency before. For each target source, we give the flux density in S- and X-bands as well as the number of AUSTRAL sessions that source was successfully observed in. Some sources were scheduled but not detected (n/d) in the analysis

With the development of the new scheduling mode for combined sessions with small and large telescopes (see Sect. 3.2), we are confident that in the future, the AUSTRALs will take even more responsibility in monitoring sources in the southern sky.

## Outlook

After a break in mid 2015, the AUSTRAL program was resumed in January 2016, with one session scheduled per month. The current emphasis is on technical development and observational improvements. This includes further optimisation of the scan length through the implementation of elevation dependent SEFD values in the scheduler and seeking improvements in physical performance and reliability. At the moment, we are working on a new observing mode, enabling data rates up to 2 Gbps using the current hardware. The initial tests revealed schedules with up to 60 scans per hour using the new mode, a number that could be even further improved when the calibration times are shortened. More automation from scheduling through to correlation is a major priority of the AuScope VLBI network. There is ongoing work on a dynamic scheduling module, allowing for a flexible and automated participation in observations (Lovell et al. 2016). This would allow for significantly more (and possibly continuous) observations at much lower operational cost per session. Soon, all AuScope stations will be equipped with local RAID systems, allowing alternative and more flexible data recording and data transport. Plans for high-speed internet connections are also under way.

All three AuScope antennas will be equipped with broadband VGOS receivers by mid 2017. This will enable future AUSTRAL sessions to continue their trailblazing observations, this time in full VGOS mode.

## Summary

The AUSTRAL VLBI program is an independent, regional VLBI observing program, coordinated by the VLBI group at the University of Tasmania. Using a network of small and fast radio telescopes, the AUSTRAL sessions realise a new style of observing, more aligned with the goals of VGOS. In this paper, we have presented some general statistics of these observations and described how a high cadence observing program paired with targeted research enabled a significant improvement in the number of observations and results. We find that a comprehensive understanding of all aspects of the observations, which was achieved in the course of this program, was essential for this.

Emphasis was given to developing a proper scheduling strategy for the AUSTRAL sessions. We described the path to a mode, where the antennas spend equal time on observing and slewing, enabling up to 35 scans per hour per station. The effect of the improved schedules is reflected in the results, with baseline length repeatabilities having improved by a factor of two since the start of the program. Overall, the results of the AUSTRAL sessions are comparable with those of the standard IVS experiments in terms of absolute station positions, and are generally better in baseline length repeatabilities for the AUSTRAL baselines. Deficiencies can be found for measurements of the EOPs, where the small AUSTRAL network is clearly inferior to the global networks of the IVS R-experiments. The benefit of the AUSTRAL program for future realisations of the celestial reference frame was discussed, revealing comparable source position accuracies for sources above the AUSTRAL network to those found in global sessions. Despite the relatively low sensitivity of the network, there were also good achievements made in astrometry using the AUSTRAL-astro-sessions.

Finally, we would like to once more draw attention to the fact that the AUSTRAL program, together with other IVS sessions of these five antennas between 2011 and 2015, comprises the most dense geodetic VLBI time series yet achieved. We believe that this is a great data set for future studies concentrating on high-frequency variations in baselines, station coordinates, and EOPs, or for a more balanced (in terms of cadence) comparison with other space geodetic techniques, including the global navigation satellite systems (GNSS).

## Footnotes

1 CONT campaigns are continuous VLBI campaigns of typically 2 weeks duration which have been organised by the IVS at irregular intervals since 1994 (http://ivscc.gsfc.nasa.gov/program/cont14/).

2
http://code.google.com/p/open-monica/.

3
moprin is a development of D. Horsley, a former Ph.D. student at UTAS.

4
http://auscope.phys.utas.edu.au/opswiki.

5 Frequency ratio squared $\approx$(8.4/2.3)$^2$.
